# Serine hydroxymethyltransferase localised in the endoplasmic reticulum plays a role in scavenging H_2_O_2_ to enhance rice chilling tolerance

**DOI:** 10.1186/s12870-020-02446-9

**Published:** 2020-05-26

**Authors:** Changxun Fang, Pengli Zhang, Lanlan Li, Luke Yang, Dan Mu, Xue Yan, Zhong Li, Wenxiong Lin

**Affiliations:** 1 Fujian Provincial Key Laboratory of Agroecological Processing and Safety Monitoring, College of Life Sciences, Fujian Agriculture and Forestry University, Fuzhou, 350002 P. R. China; 2 Key Laboratory of Crop Ecology and Molecular Physiology (Fujian Agriculture and Forestry University), Fujian Province University, Fuzhou, 350002 P. R. China; 3 Key Laboratory of Ministry of Education for Genetics, Breeding and Multiple Utilization of Crops, Fujian Agriculture and Forestry University, Fuzhou, 350002 P. R. China

**Keywords:** Rice, Chilling, Serine hydroxymethyltransferase, ROS, Protein-protein interactions

## Abstract

**Background:**

Rice is a chilling-sensitive crop that would suffer serious damage from low temperatures. Overexpression of the *Lsi1* gene (*Lsi1*-OX) in rice enhances its chilling tolerance. This study revealed that a serine hydroxymethyltransferase (OsSHMT) mainly localised in the endoplasmic reticulum (ER) is involved in increasing tolerance to chilling.

**Results:**

A higher transcription level of *OsSHMT* was detected in *Lsi1*-OX rice than in the wild type. Histone H1 and nucleic acid binding protein were found to bind to the promoter region of *OsSHMT* and regulate its expression, and the transcription levels of these proteins were also up-regulated in the *Lsi1*-OX rice. Moreover, OsSHMT interacts with ATP synthase subunit α, heat shock protein Hsp70, mitochondrial substrate carrier family protein, ascorbate peroxidase 1 and ATP synthase subunit β. *Lsi1*-encoded protein OsNIP2;1 also interacts with ATP synthase subunit β, and the coordination of these proteins appears to function in reducing reactive oxygen species, as the H_2_O_2_ content of transgenic *OsSHMT Arabidopsis thaliana* was lower than that of the non-transgenic line under chilling treatment.

**Conclusions:**

Our results indicate that ER-localised OsSHMT plays a role in scavenging H_2_O_2_ to enhance the chilling tolerance of *Lsi1*-OX rice and that ATP synthase subunit β is an intermediate junction between OsNIP2;1 and OsSHMT.

## Background

Plants require a given temperature range for normal growth and development, and sudden changes in temperature can cause growth inhibition and even death [1]. Cold spells in late spring are examples of sudden drops in temperature over a short time and can adversely affect the growth of rice. Some varieties of rice, mainly those in the *indica* subspecies, are particularly sensitive to low temperatures [2, 3]. Continuous chilling of crop plants has been shown to result in increased reactive oxygen species (ROS) and cell membrane peroxidation and to affect the expression of chloroplast genes, inhibiting photosynthesis [4]. Such ROS include O^2−^, H_2_O_2_ and OH^−^ [5].

Fang et al. [6] documented that chilling treatment inhibited the expression of chlorophyll synthesis genes and promoted the expression of proteasome genes from temperature-sensitive Dular rice (*Oryza sativa ssp. indica*), leading to chloroplast damage, chlorophyll degradation, loss of green colour and partial degradation of RNA in leaves. Cui et al. [7] found that the whitening of Dular leaves under chilling stress was associated with the deletion of the gene coding sequence of pentatricopeptide (LOC_Os09g29825.1), which is an RNA-binding protein, and the gene coding sequence missed 8 bases relative to that from Nipponbare rice, resulting in a frameshift mutation of the coding sequence and deactivation of the protein function. The mutant gene was named *DUA1*. Inactivation of the DUA1 protein in Dular rice leads to a loss of RNA editing capacity under chilling stress, resulting in rice seedlings that exhibit chloroplast development defects and leaf chlorosis. When the silicon-absorbing gene (*Lsi1*) was over-expressed in Dular rice, the chilling tolerance of the transgenic line was significantly improved, and the leaves maintained their fresh green colour under chilling treatment. The expression of genes from the photosynthesis pathway of transgenic rice was enhanced under low temperature stress, and the expression of genes involved in the proteasome was down regulated. In addition, the transcription level of the gene encoding serine hydroxymethyltransferase (SHMT, *LOC_Os03g52840*) was up-regulated in the transgenic rice, but down-regulated in the wild-type, and the expression of its corresponding miRNA changed in an opposite way, indicating that OsSHMT may be involved in regulating the chilling-tolerance of Dular [6].

SHMT is widely distributed in plants [8]. The enzyme catalyses the reversible exchange between serine and glycine (glycine CH_2_-THF H_2_O↔serine THF) [9, 10], and these two amino acids are precursors of chlorophyll, tryptophan and ethanolamine [11]. Therefore, SHMT plays an important role in the photorespiration processes of plants [12]. Studies have found that a mitochondrial serine hydroxymethyltransferase gene mutation in rice (*osshm1*) causes blockage of the photorespiration pathway, thereby affecting the Calvin cycle and the efficiency of light energy; excess light in the chloroplast then leads to the accumulation of ROS, resulting in disruption of chloroplast development and fewer and smaller chloroplasts with less grana [13].

In addition, SHMT plays a positive role in regulating plants’ resistance to stress. In *Arabidopsis*, expression of NADH dehydrogenase was shown to be inhibited by chilling, leading to the continuous accumulation of ROS, suppressing the transcription of cold-response genes and hypersensitivity to chilling [14]. SHMT activity contributes to a reduction of ROS accumulation in the chloroplast and therefore reduces oxidative damage [15]; as a second messenger, a reduction in ROS would also prevent the transmission of the chilling signal and reduce the damage caused by ROS. The recessive mutation *shmt1–1* in *Arabidopsis* results in abnormal regulation of cell death, leading to chlorotic and necrotic diseases under various environmental conditions, and mutants that carry the *shmt1–1* allele exhibit more H_2_O_2_ accumulation than wild-type plants under salt stress, resulting in greater chlorophyll loss [16]. To the best of our knowledge, the specific role of OsSHMT in the regulation of rice cold resistance has not been reported.

In this study, an *OsSHMT* gene (*LOC_Os03g52840*) from rice was amplified, the subcellular localisation of OsSHMT was investigated, and proteins that bind to the promoter region of *OsSHMT* were obtained. The proteins that interact with OsSHMT were also investigated to indicate the regulation network of OsSHMT in rice under chilling stress, revealing the possible role of OsSHMT in scavenging H_2_O_2_ to improve cold resistance in rice.

## Results

### Chilling tolerance of Dular and *Lsi1*-OX rice

**Fig. 1:**
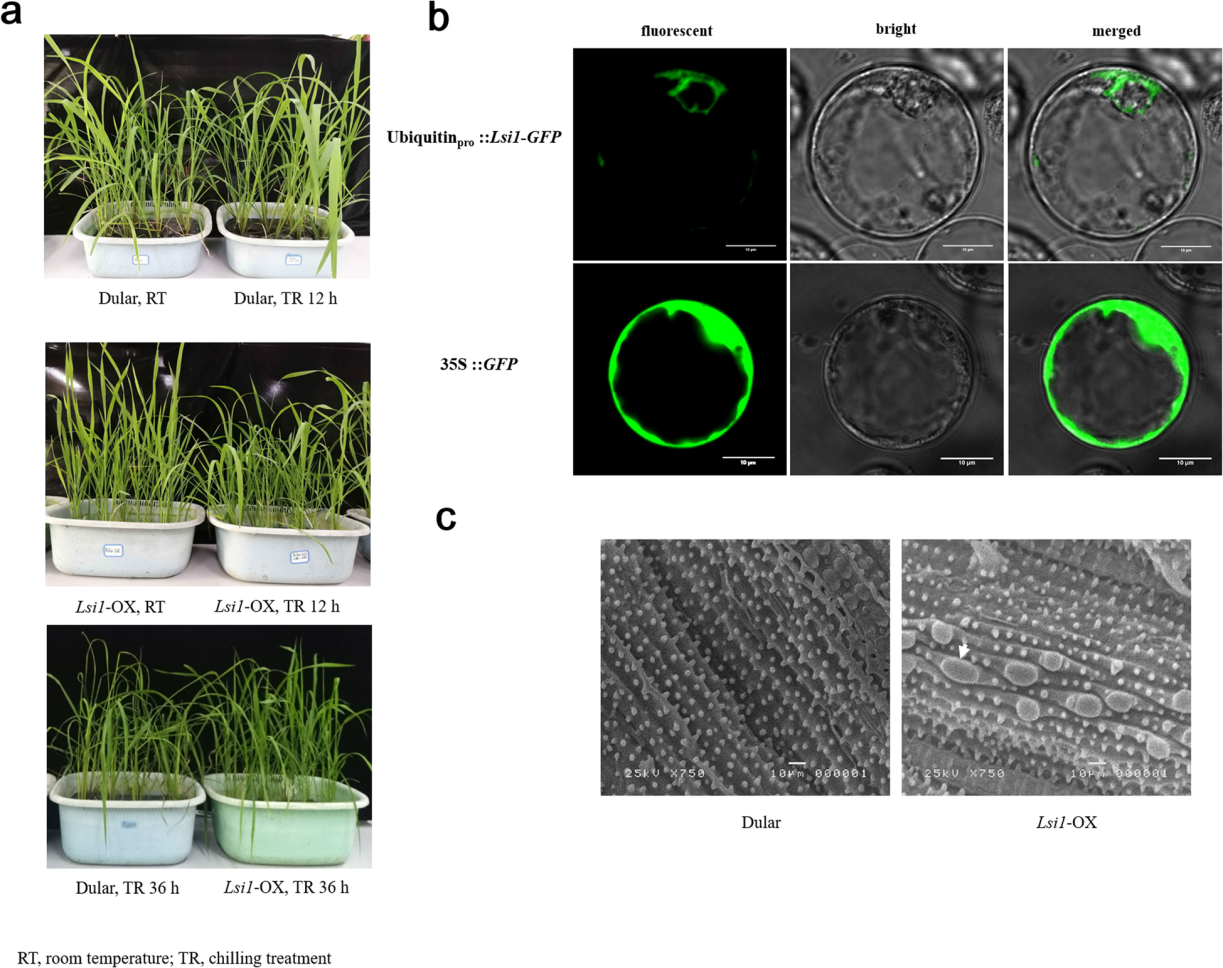
Phenotypic changes in the leaves under chilling treatment (**a**), subcellular localisation of *Lsi1* driven by ubiquitin promoter (**b**) and SEM images of the leaf surface of Dular and *Lsi1*-OX (**c**). The Dular rice and the transformed *Lsi1*-OX line were treated at 4 °C for 36 h and the phenotypic changes in the leaves of the two rice lines were compared (**a**). *Lsi1* was fused with GFP and inserted into a modified pCambia 1301 vector in which the *Lsi1* gene was transcribed by the ubiquitin promoter. The recombinant *Lsi1* expression vector was transformed in the rice protoplast and subcellular localisation of OsNIP2;1 was detected using laser scanning confocal microscopy (**b**). SEM images of the surfaces of the two rice leaves were compared to determine the differences in silica deposition (**c**)

The Dular and *Lsi1*-OX transgenic lines presented different tolerances to the chilling treatment of 4 °C for 36 h. Most of the leaves from the Dular rice became whiter, whilst the *Lsi1*-OX line maintained tolerance to chilling (Fig. 1a). Further studies showed that the *Lsi1* overexpression vector was driven by the ubiquitin promoter, and the translate OsNIP2;1 was localised in the cytoplasm (Fig. 1b), which differs significantly from the original localisation in the cell membrane [17]. This result suggests that OsNIP2;1 may have more roles than its initial function, which belongs to the aquaporin family and controls silicon accumulation in rice [17]. Scanning electron microscope (SEM) images of the leaves revealed that silica bodies in the *Lsi1*-OX rice leaves were bigger than those of the Dular rice (Fig. 1c).

### Subcellular localisation of OsSHMT

**Fig. 2:**
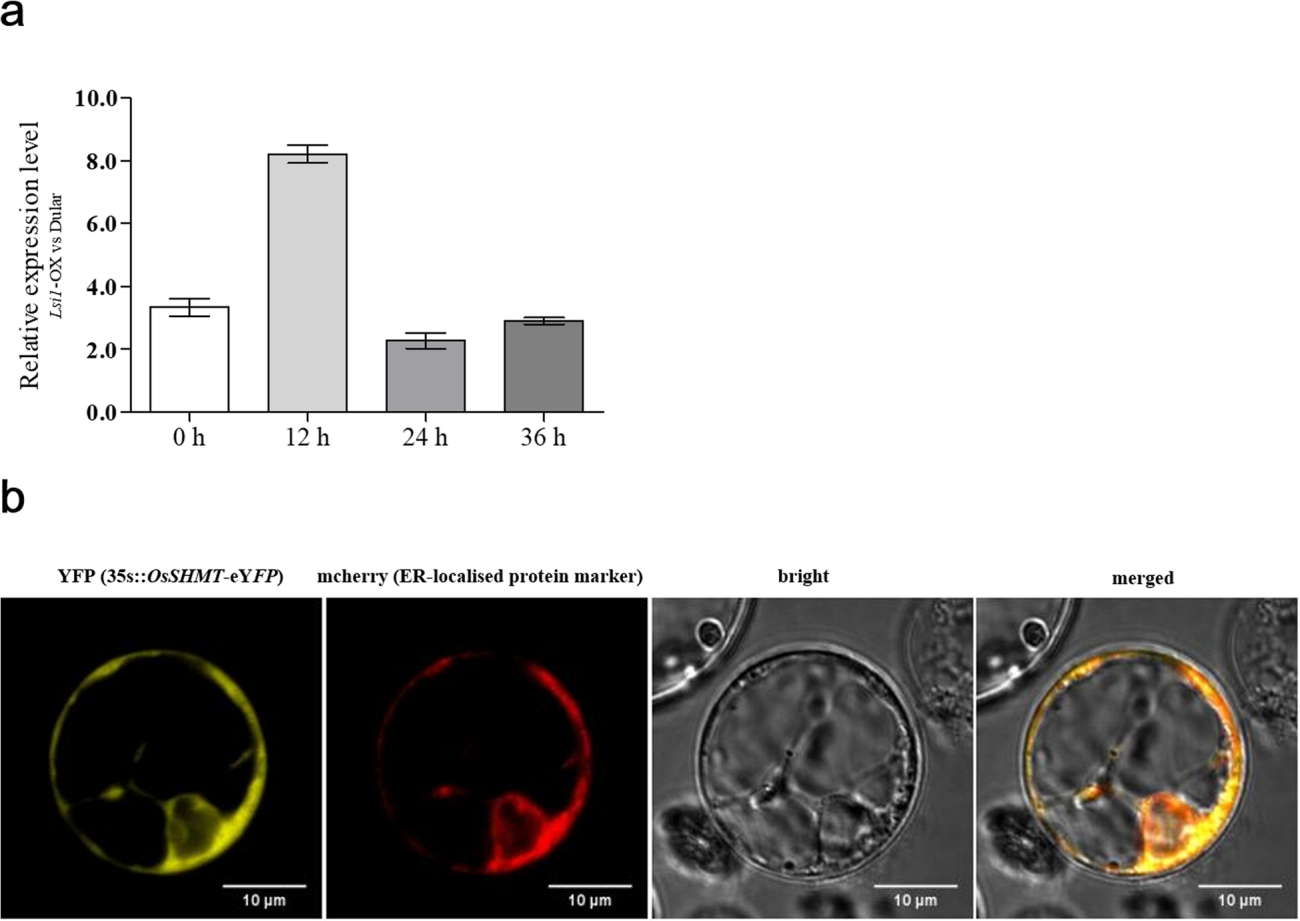
Relative expression of *OsSHMT* in the *Lsi1*-OX rice line and Dular rice (**a**) and subcellular co-localisation of OsSHMT protein with endoplasmic reticulum marker protein (**b**). Changes in the gene expression level of *OsSHMT* before chilling treatment and after treatment for 12, 24 and 36 h were compared for *Lsi1*-OX and Dular rice (**a**). *OsSHMT* was fused with eYFP and inserted into pCambia 2300 to construct the recombinant vector for *OsSHMT* expression and detect subcellular protein localisation. The recombinant vector was transformed into rice protoplast for transient expression, together with a mcherry fused ER localised protein as a marker. Yellow fluorescence and mcherry fluorescence were respectively detected and then merged to validate the OsSHMT subcellular localisation (**b**)

Overexpression of *Lsi1* in Dular rice results in changes in the expression of thousands of genes [6], including the *OsSHMT* gene. Expression of *OsSHMT* was up-regulated in the *Lsi1*-OX rice in comparison with the Dular rice (Fig. 2a). Subcellular localisation of OsSHMT (LOC_Os03g52840) in the rice protoplast then showed that yellow fluorescence was concentrated around the nucleus, and no obvious endonuclear fluorescence was observed, whereas significant yellow fluorescence was seen in the whole nucleus in the rice protoplast transformed with the eYFP vector (Fig. S1). This suggested that OsSHMT was localised on the endoplasmic reticulum around the nucleus. Further co-localisation of the endoplasmic reticulum marker protein with mcherry indicated that OsSHMT was widely distributed in the endoplasmic reticulum, as the mcherry fluorescence from the marker protein and yellow fluorescence from OsSHMT infused with eYFP was completely overlapping (Fig. 2b). OsSHMT is involved in the photorespiratory processes of plants, and existing studies have suggested that the protein is localised to mitochondria, chloroplasts and cytoplasm. The results of this study complement those findings.

### Promoter region of *OsSHMT* and the binding proteins

**Fig. 3:**
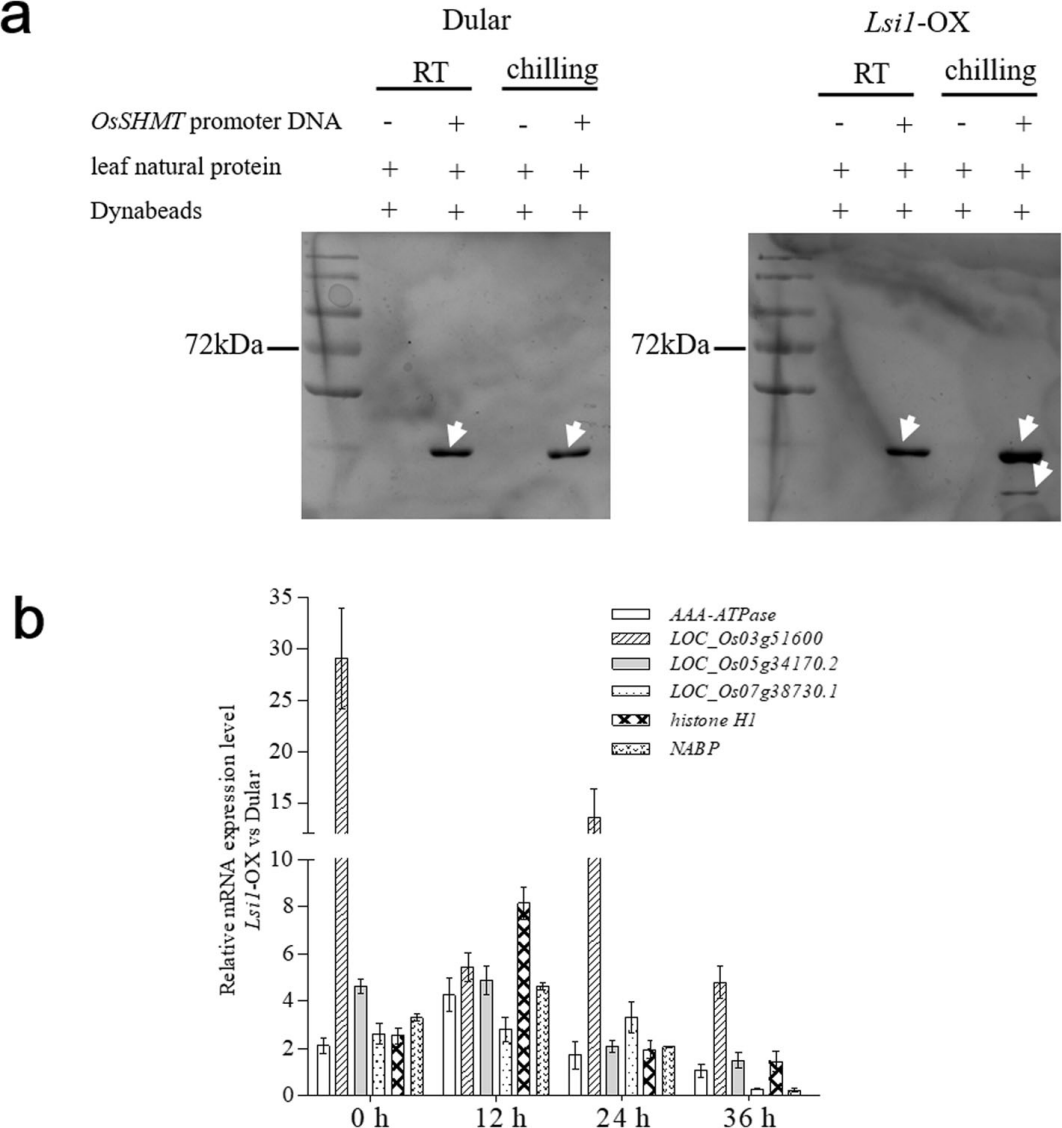
Proteins binding on the *OsSHMT*-promoter in Dular and *Lsi1*-OX rice and the differences in transcription level between the two types of rice. The promoter region of *OsSHMT* was amplified using the specific primers with biotin labelled at the 5′ end and then fused with Streptavidin-coupled Dynabeads and natural leaf proteins from Dular or *Lsi1*-OX rice. The protein and DNA complex mixture was extracted and incubated with *OsSHMT* promoter-containing Dynabeads and fished using a magnetic frame to collect the proteins and then separated by SDS-PAGE (**a**). qPCR was conducted to determine the change in the transcription levels of AA-ATPase, histone H1, nucleic acid binding protein (NABP) and tubulin/FtsZ domain containing protein (LOC_Os03g51600, LOC_Os05g34170, LOC_Os07g38730) for *Lsi1*-OX and Dular rice under chilling treatment of different durations (**d**)

The promoter region 2597 bp upstream of the CDS of the *OsSHMT* gene from Dular rice was amplified and labelled with biotin at the 5′ flanking region of this DNA fragment (Table S1). According to the DNA pull-down results, compared with the control group at room temperature, chilling treatment of *Lsi1*-OX induced more proteins to bind to the promoter region of *OsSHMT*. In contrast, chilling treatment of the Dular rice had no significant effect on the proteins binding to the promoter (Fig. 3a). Identification of the proteins showed that retrotransposon protein (Ty3-gypsy subclass), glyceraldehyde-3-phosphate dehydrogenase, and AT hook motif protein were identified from both the *Lsi1*-OX and Dular rice under chilling treatment or room temperature conditions; however, some proteins, such as histone H1, nucleic acid binding protein (NABP) and tubulin/FtsZ domain-containing protein (LOC_Os03g51600.1) were only identified from the chilling-treated *Lsi1*-OX group; another tubulin/FtsZ domain-containing protein (LOC_Os05g34170.2) was identified from the chilling-treated *Lsi1-*OX and Dular groups and from the Dular group at room temperature. AAA-type ATPase family protein was identified from the Dular group at room temperature but not from the chilling-treated Dular group, and this protein was induced in the chilling-treated *Lsi1*-OX group in comparison with its control group at room temperature (Table 1). The tubulin/FtsZ domain-containing proteins (LOC_Os03g51600.1, LOC_Os05g34170.2, LOC_Os07g38730.1), histone H1 and NABP were selected to analysis their gene expression level on the two rice. A comparison of the gene transcription levels in Dular and *Lsi1*-OX rice after chilling treatment for 12, 24 and 36 h in comparison with 0 h, revealed opposite trends in the two rice lines, and the gene expression level was up-regulated in the *Lsi1*-OX rice in comparison with Dular rice under the same treatment conditions (Fig. 3b). The results indicate that these genes act in combination to exert a positive role in the regulation of *OsSHMT* expression.

**Table 1:** Proteins binding on the *OsSHMT* gene promoter from Dular and *Lsi1*-OX

Protein ID	Unique peptide number	Unique spectra number	Coverage	Description
Dular, RT				
LOC_Os03g58470.1	11	121	0.3481	retrotransposon protein, putative, Ty3-gypsy subclass, expressed
LOC_Os04g58730.1	4	6	0.1527	AT hook motif domain containing protein, expressed
LOC_Os03g03720.1	4	4	0.1261	glyceraldehyde-3-phosphate dehydrogenase, putative, expressed
LOC_Os12g37260.1	3	3	0.0434	lipoxygenase 2.1, chloroplast precursor, putative, expressed
LOC_Os11g47970.1	3	3	0.0687	AAA-type ATPase family protein, putative, expressed
LOC_Os04g42320.1	3	3	0.0414	AT hook motif family protein, expressed
LOC_Os07g08710.1	3	3	0.1871	AT hook-containing DNA-binding protein, putative, expressed
Dular, chilling				
LOC_Os03g58470.1	10	130	0.3311	retrotransposon protein, putative, Ty3-gypsy subclass, expressed
LOC_Os04g58730.1	4	8	0.1527	AT hook motif domain containing protein, expressed
LOC_Os07g08710.1	4	9	0.223	AT hook-containing DNA-binding protein, putative, expressed
LOC_Os05g34170.2	4	7	0.1036	tubulin/FtsZ domain containing protein, putative, expressed
LOC_Os03g03720.2	3	4	0.1226	glyceraldehyde-3-phosphate dehydrogenase, putative, expressed
*Lsi1*-OX, RT				
LOC_Os03g58470.1	10	145	0.3447	retrotransposon protein, putative, Ty3-gypsy subclass, expressed
LOC_Os04g42320.1	6	9	0.0674	AT hook motif family protein, expressed
LOC_Os01g72049.1	5	5	0.1975	retrotransposon, putative, centromere-specific, expressed
LOC_Os05g34170.2	3	4	0.0811	tubulin/FtsZ domain containing protein, putative, expressed
LOC_Os07g38730.1	2	2	0.0444	tubulin/FtsZ domain containing protein, putative, expressed
*Lsi1*-OX, chilling				
LOC_Os03g58470.1	15	205	0.3823	retrotransposon protein, putative, Ty3-gypsy subclass, expressed
LOC_Os05g51850.1	8	8	0.1413	AT hook-containing DNA-binding protein, putative, expressed
LOC_Os04g58730.1	7	10	0.2506	AT hook motif domain containing protein, expressed
LOC_Os11g47970.1	6	6	0.1309	AAA-type ATPase family protein, putative, expressed
LOC_Os01g72049.1	6	6	0.1975	retrotransposon, putative, centromere-specific, expressed
LOC_Os04g42320.1	6	7	0.0721	AT hook motif family protein, expressed
LOC_Os07g08710.1	5	9	0.2662	AT hook-containing DNA-binding protein, putative, expressed
LOC_Os04g49990.1	4	5	0.2507	AT hook motif domain containing protein, expressed
LOC_Os05g34170.2	4	5	0.1059	tubulin/FtsZ domain containing protein, putative, expressed
LOC_Os08g40150.1	3	3	0.1102	AT hook motif domain containing protein, expressed
LOC_Os04g38600.2	3	3	0.1111	glyceraldehyde-3-phosphate dehydrogenase, putative, expressed
LOC_Os06g04020.1	3	3	0.1458	histone H1, putative, expressed
LOC_Os03g52490.1	2	2	0.098	nucleic acid binding protein, putative, expressed

### Proteins interacting with OsSHMT

**Fig. 4:**
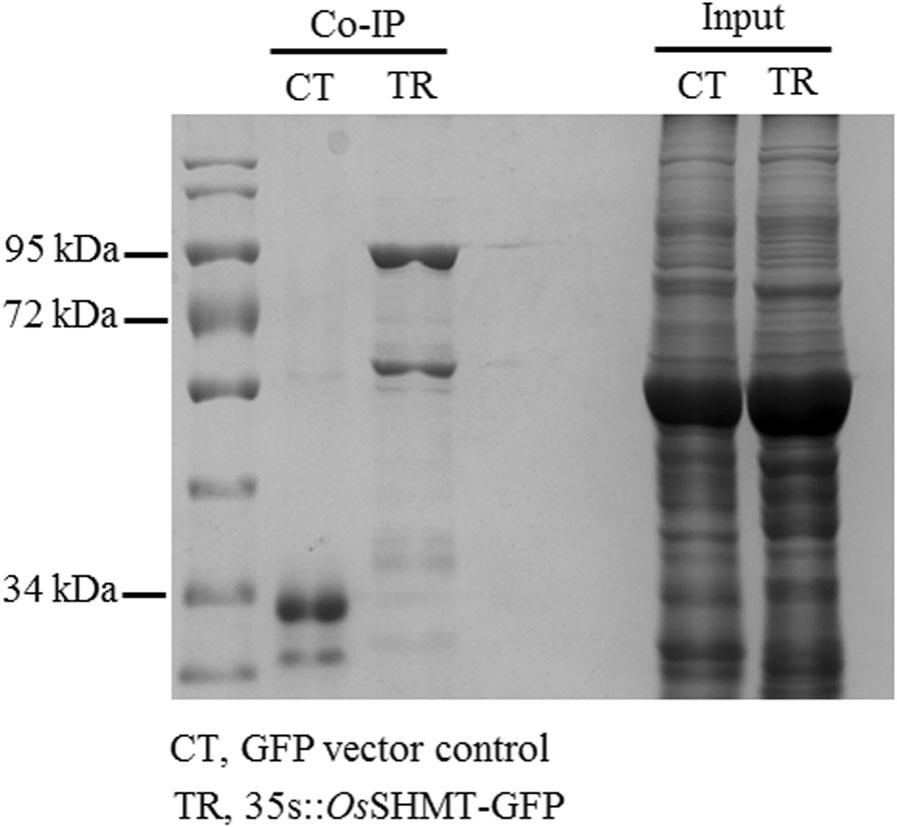
Protein interactions with OsSHMT in *Arabidopsis thaliana.* The recombinant vector of *OsSHMT* fused with GFP was used to genetically transform *Arabidopsis thaliana* and T_3_ homozygous transgenic lines were taken to isolate proteins interacting with OsSHMT. A vector containing only GFP without *OsSHMT* was used as a control to transform into *A. thaliana* to obtain the transformed lines. Natural leaf proteins from *OsSHMT* transgenic *A. thaliana* and GFP transgenic *A. thaliana* were respectively extracted and incubated with GFP-Trap agarose beads to reveal the proteins interacting with OsSHMT

**Fig. 5:**
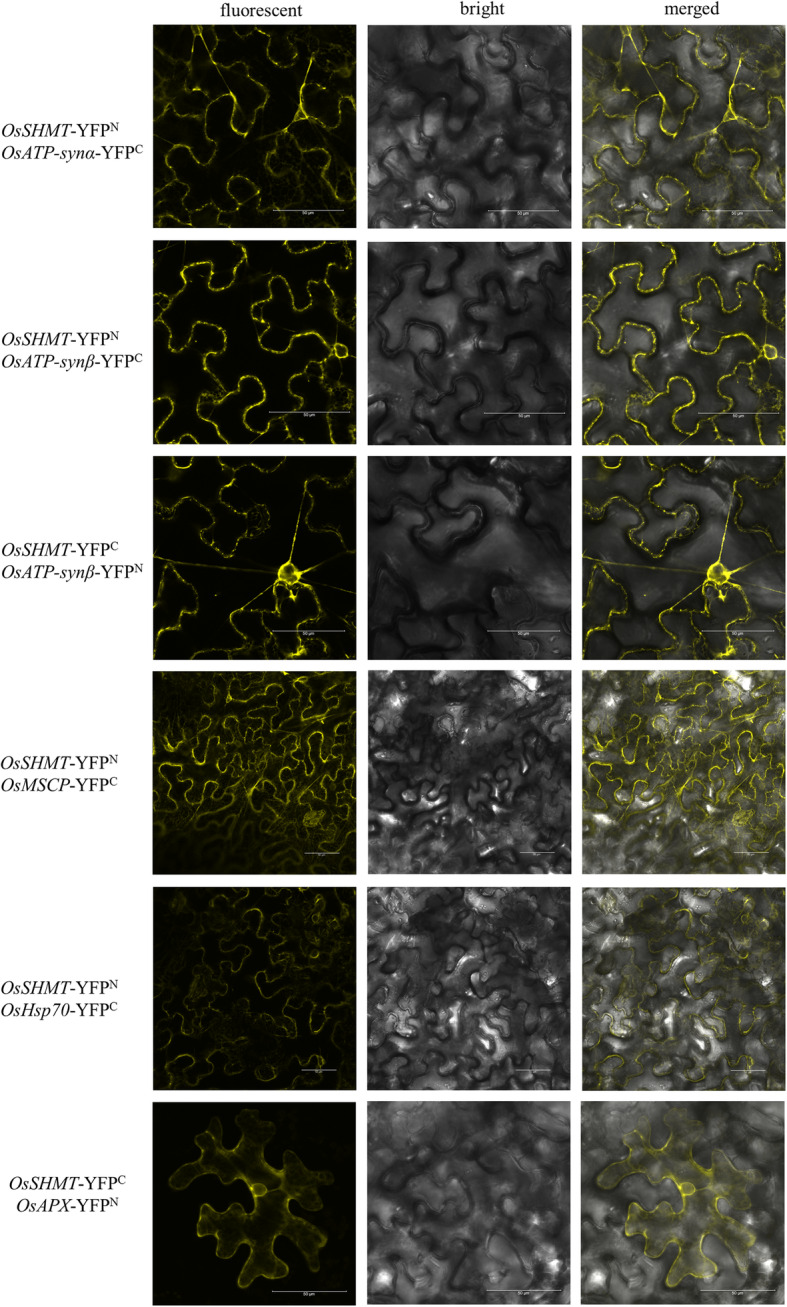
Bimolecular fluorescence complementation validation of the bio-interaction of OsSHMT and ATP synthase subunit α, ATP synthase subunit β, Hsp70, MSCP and APX from rice. To validate the positive interaction of OsSHMT with ATP synthase α subunit (ATP-synα), ATP synthase β subunit (ATP-synβ), heat shock protein 70, mitochondrial substrate carrying family (MSCP) protein E and ascorbate peroxidase (APX) in rice, their genes were amplified from Dular rice and respectively infused with the N-terminal or C-terminal of YFP to construct YFP^n^- and YFP^c^-containing recombinant vectors for bimolecular fluorescence complementation (BiFC), according to transient transformation in the leaves of *Nicotiana benthamiana.* The YFP fluorescence was detected using laser confocal microscopy under 488-nm excitation light

Based on the GFP-TRAP method to obtain the proteins interacting with OsSHMT, it was found that several proteins co-precipitated with OsSHMT, in comparison with the GFP-vector control (Fig. 4; Table S2). Some of these proteins, including ATP synthase α subunit, ATP synthase β subunit, heat shock protein 70, mitochondrial substrate carrying family protein E, ascorbate peroxidase 1, are defense proteins that interact with OsSHMT protein (Table 2). Further determination of the interaction of OsSHMT with ATP synthase subunit α, ATP synthase subunit β, Hsp70, MSCP and APX from the rice showed that yellow fluorescence was detected in the leaves of tobacco infected with OsSHMT and each of these proteins respectively. No fluorescence was detected in the control group, demonstrating that OsSHMT positively interacts with the above proteins (Fig. 5). The results indicate that OsSHMT interacted with defence-related proteins in rice, including APX, MSCP, HSP70 and ATP synthase, to jointly regulate chilling resistance.

**Table 2:** The target protein from *rdr6* interacted with *Os*SHMT identified by LC-MS

Accession	Description	Sum PEP Score	Coverage	Peptides	PSMs	Unique Peptides
ATCG00480.1	ATP synthase subunit beta	39.443	50.60241	19	35	18
ATCG00120.1	ATP synthase subunit alpha	30.137	26.82446	13	28	11
AT1G07890.2	ascorbate peroxidase 1	9.281	37.6	7	8	7
AT5G02490.1	Heat shock protein 70 (Hsp 70) family protein	15.568	9.035222	5	7	5
AT5G46800.1	Mitochondrial substrate carrier family protein	5.09	10	4	4	4
ATCG00480.1	ATP synthase subunit beta	6.277	8.634538	3	3	3

### H_2_O_2_ content in the *OsSHMT* transgenic *A. thaliana* and wild type

**Fig. 6:**
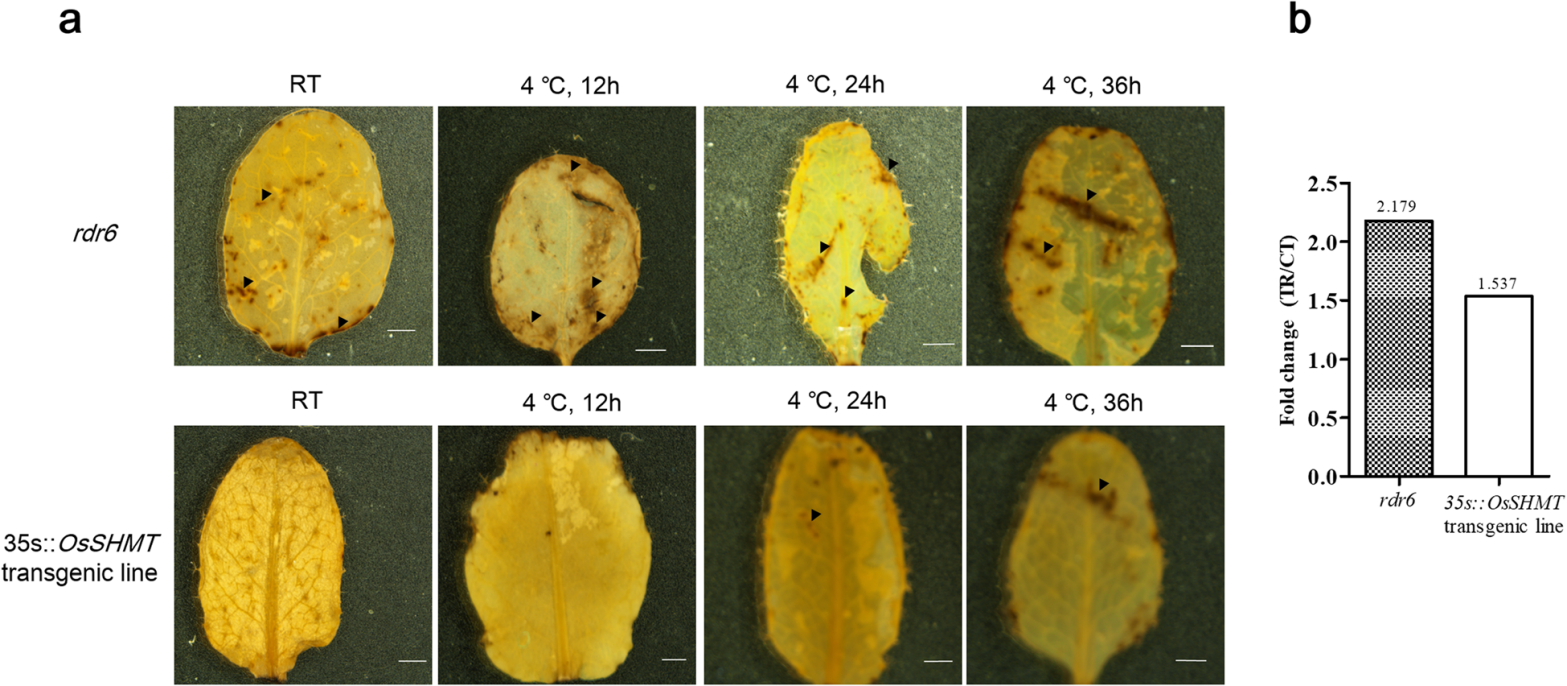
DAB staining of leaves of *OsSHMT* transgenic and wild type *A. thaliana* (**a**) and the H_2_O_2_ content in the leaves (**b**). *OsSHMT* transgenic *Arabidopsis thaliana* and its wild-type were exposed to 0 °C for 12, 24 and 36 h, and leaves were sampled and stained with DAB, after which the chlorophyll in the leaves was dissolved using ethanol, leaving dark brown precipitate on the leave. Arrowheads indicate reddish-brown spots. Bar = 1 mm. The H_2_O_2_ content in the leaves was determined using an H_2_O_2_ determination kit (Solarbio Life Sciences)

To further indicate the function of OsSHMT in scavenging H_2_O_2_, wildtype *A. thaliana* and positive transgenic T_3_*A. thaliana* seedlings were exposed to a temperature of 4 °C for 12, 24 and 36 h. The leaves of the wild type showed more reddish-brown spots after Diaminobenzidine (DAB) staining. The transgenic line also showed reddish-brown spots, but the spots were small in size and number (Fig. 6a). Determination of the leaf H_2_O_2_ content of the transgenic line and wild type of *A. thaliana* showed that the increase in H_2_O_2_ in the transgenic line of *A. thaliana* after chilling treatment was significantly lower than that of the wild type that underwent the same treatment (Fig. 6b).

### OsNIP2;1 interacts with ATP synthase subunit β from rice

**Fig. 7:**
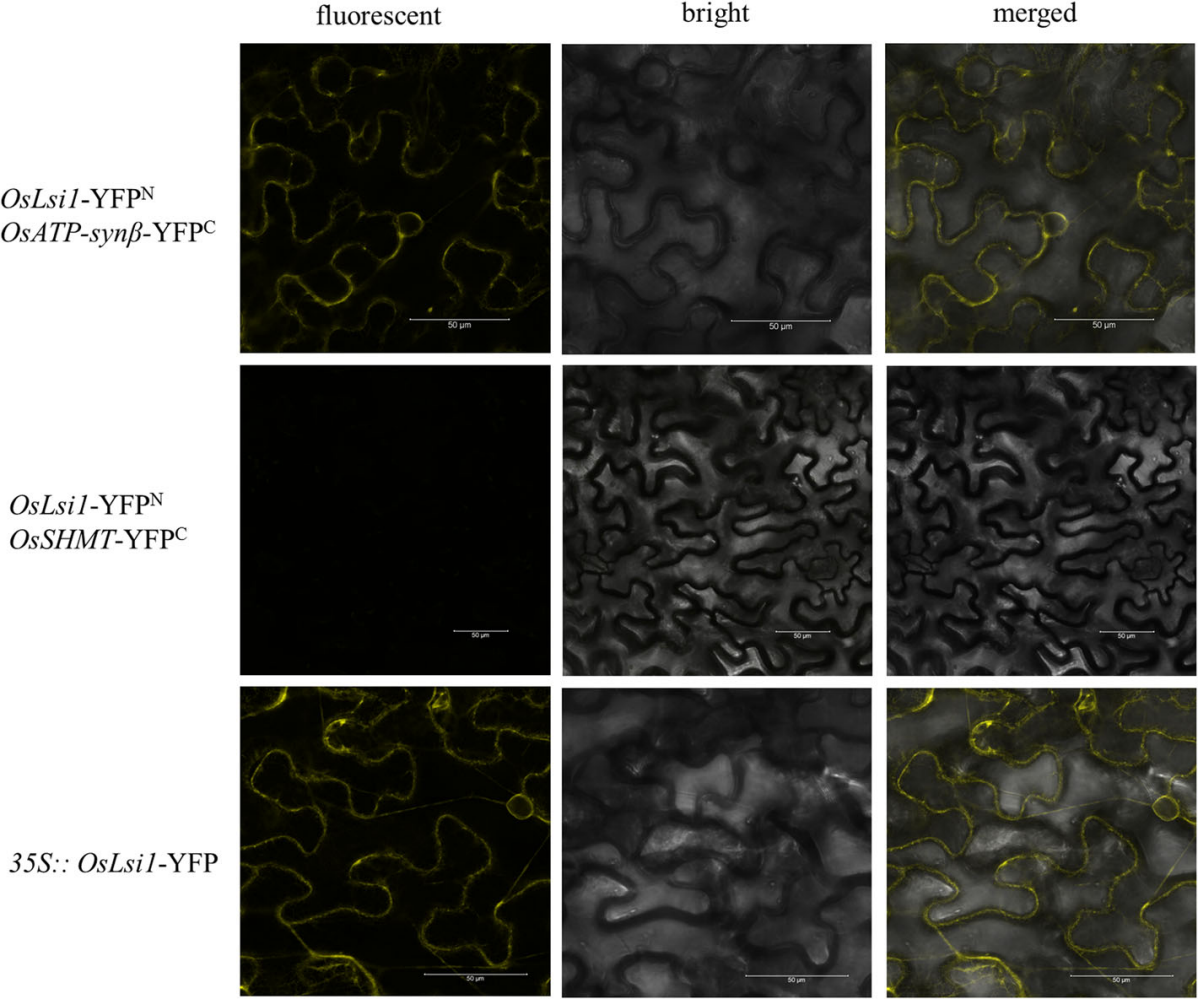
Bimolecular fluorescence complementation validates the bio-interaction of *O*sNIP2;1 and ATP-synβ. *Lsi1* was infused with the N-terminal domain of YFP and ATP synthase subunit β and OsSHMT was infused with the C-terminal domain of YFP. *Lsi1* with N-terminal domain was then co-expressed with ATP synthase subunit β or OsSHMT with the C-terminal domain of YFP, respectively, in the leaves of *Nicotiana benthamiana* for 48 h, and YFP fluorescence was detected using laser confocal microscopy under 488-nm excitation light. The subcellular localisation of *Lsi1* was also detected in the leaves of *Nicotiana benthamiana*

*Lsi1* encodes the aquaporins protein OsNIP2;1, which is a nodulin 26-like intrinsic protein (NIP) and is localised in the membrane of the cell. Overexpression of *Lsi1* in Dular rice using a ubiquitin promoter resulted in the cytoplasm localisation of this protein, enabling OsNIP2;1 to play multiple roles. The interaction between OsNIP2;1 and OsSHMT was also investigated. The results showed no direct interaction between OsNIR2;1 and OsSHMT. However, OsNIP2;1 interacted with ATP-synβ, a protein that also interacts with OsSHMT, and the results indicate that ATP-synβ acts as an intermediate junction between OsNIP2;1 and OsSHMT (Fig. 7).

## Discussion

Plant suffers from low-temperature frequently results in the accumulation of ROS, which would lead to lipid peroxidation of the cell membrane. Our studies here indicated that OsSHMT functions in scavenging H_2_O_2_ in the plant. OsSHMT has been declared to be localised in the mitochondria, chloroplast, cytoplasm, nucleus, plasma membrane and cytosol [13, 18, 19], and participates in the photorespiration pathway. Besides, our present study indicated that OsSHMT is also localised in the endoplasmic reticulum, which is the main organelle for protein and lipid synthesis, membrane biogenesis, xenobiotic detoxification and cellular calcium storage [20]. The activity of OsSHMT in scavenging H_2_O_2_ is considered to be prominent when the rice undergoes chilling stress.

With scavenging of H_2_O_2_, OsSHMT was found to be interacted with a couple of proteins. Among these interacted proteins, APX is the one with ROS-scavenging activity. In *Arabidopsis thaliana*, the cytosolic ascorbate peroxidase 1 (APX1) is a central component of the H_2_O_2_-scavenging system, absence of APX1 in the knock-out lines lead to the collapse of the chloroplast H_2_O_2_ clearance system, thereby increasing levels of H_2_O_2_ and oxidative proteins [21]. The heat shock protein Hsp70 also interacts with OsSHMT. Hsp70 is a molecular chaperone protein and plays a critical role in stress tolerance [22]; in rice, chloroplast-localised Hsp70 is essential for chloroplast development under high-temperature conditions, whilst overexpression of mitochondrial HSP70 in rice suppresses programmed cell death [23, 24]. It is therefore suggested that the multiple functions of Hsp70 contribute to enhancing rice chilling tolerance. As ATP is required in the catalytic reaction to scavenging ROS, OsSHMT also interacts with subunit α and subunit β of ATP synthase. These subunits are widely distributed in mitochondria and chloroplasts, and the synergistic effect of subunits function in produced ATP from ADP in the presence of a proton gradient across the membrane, which is generated by electron transport complexes of the respiratory chain [25], and the mitochondrial substrate carrier family proteins catalyse the passage of hydrophilic compounds such as ADP/ATP across the inner mitochondrial membrane [26]. The coordination of these proteins appears to provide ATP for OsSHMT activating in scavenging H_2_O_2._

As the dominant role of OsSHMT in scavenging H_2_O_2_, increasing expression level of *OsSHMT* contributes to enhance its capacity. Gene expression of *OsSHMT* was higher in the *Lsi1*-OX rice than that in its wild-type Dular [6]. The different expression level of *OsSHMT* from these two rice lines mainly attributes to the expression of transcription regulators to *OsSHMT*. NABP and histone H1 were two transcription regulators binding on the promoter of *OsSHMT*. NABP has a dual role in regulation of gene expression at the transcriptional and posttranscriptional levels [27, 28]. Histones in eukaryotes are structural proteins that bind to DNA to construct chromatin nucleosomes. Histone H1 is commonly considered a transcriptional repressor because it prevents transcription factors and chromatin remodelling complexes from entering DNA [29]. Some other proteins, including AAA-ATPase family proteins and tubulin/FtsZ domain containing protein, were co-occurrence in these DNA-binding proteins. Tubulin is the main component of plant microtubules, which plays an important part in the regulation of stress tolerance and thus responds to a variety of intracellular and external stimuli [30–32]. Higher transcriptional levels of these genes in the *Lsi1*-OX rice than those in Dular, suggesting that these factors have a positive impact in regulating the expression of *OsSHMT* gene. Whether these proteins are precisely or indirectly linked to the *OsSHMT* gene promoter remains to be revealed.

Overexpression of *Lsi1* in the Dular results in the endoplasmic reticulum localisation of *Lsi1*-encoded Nod26-like intrinsic protein (OsNIP2;1). Since OsSHMT is also localised on the endoplasmic reticulum, the interactions of ATP synthase β subunit respectively with *Os*NIP2;1 and OsSHMT, indicating that OsNIP2;1 can indirectly cooperate with OsSHMT through the ATP synthase β subunit, and that OsSHMT interacts with defence and anti-oxidation related proteins to regulate the chilling resistance of rice and ensure its normal growth and development. The increase in OsSHMT expression and its constructive role in scavenging H_2_O_2_ provides partial assistance to rescue the loss of chilling resistance in Dular rice due to the inactivation of *DUA1*.

## Conclusions

**Fig. 8:**
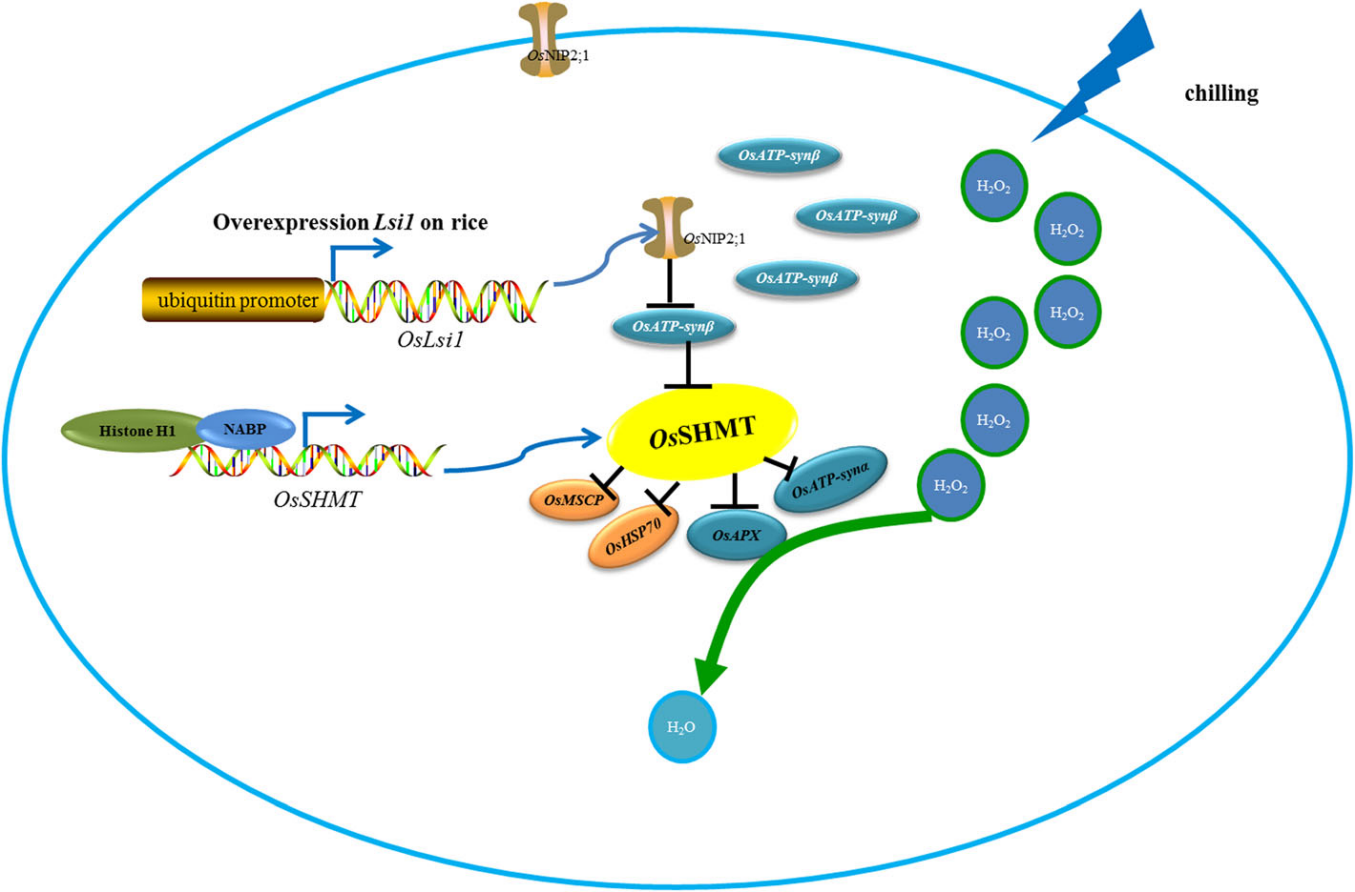
Schematic summary of the role of OsSHMT in the regulation of rice chilling tolerance. OsSHMT interacts with APX, HSP70, MSCP, ATP-synα and ATP-synβ to scavenge H_2_O_2_. ATP-synβ also interacts with OsNIP2;1 in the cytoplasm when the ubiquitin promoter is used to overexpress *Lsi1* in the rice

Overexpression of *Lsi1* in chilling-sensitive Dular rice resulted in the plasma membrane-localized OsNIP2;1 protein expressed in the cytoplasm, which would enable OsNIP2;1 to perform multiple roles. The expression of *Os*SHMT was up-regulated in the *Lsi1*-OX line in comparison with the Dular line. The differential gene expression level of *OsSHMT* may be transcriptionally regulated by NABP, and histone H1. In addition, OsSHMT interacts with APX, Hsp70, ATP-synα, ATP-synβ and MSCP to scavenge H_2_O_2,_ and ATP-synβ interacts with OsNIP2;1. Even OsSHMT does not directly interact with OsNIP2;1, ATP synthase subunit β is an intermediate junction between OsNIP2;1 and OsSHMT (Fig. 8).

## Methods

### Plant materials and treatments

In this study, Dular rice (*Oryza sativa L. subsp*. *indica*) and its transgenic line with *Lsi1* overexpression (*Lsi1*-OX) were used; Dular rice is saved at Fujian Agriculture and Forestry Universtiy (Fuzhou, China) and the *Lsi1*-OX transgenic line of Dular was germinated in our previous studies [33]. *Arabidopsis thaliana* with an RNA-dependent RNA polymerase 6 gene mutation (*rdr6*) [34] and *Nicotiana benthamiana* [35] were also used in this study.

The Dular and *Lsi1*-OX rice seeds were sterilised with 25% sodium hypochlorite solution (W/V) for 30 min and washed with sterile water to remove any residues. The sterilised seeds were soaked overnight at 30 °C in an incubator. The seeds were germinated and sown in black pots filled with a hydroponic nutrient solution suspended in a polyethylene mesh. The pots were placed in an artificial climate chamber for 10 days, during which a temperature of 26 °C was maintained for 14 h and darkness was maintained for 10 h at 22 °C. The relative humidity in the chamber was maintained at about 85%. The nutrient solution was changed weekly, and the pH was maintained between 5.5 and 6.0 throughout the experiment.

When the rice had reached the three-leaves stage, the *Lsi1*-OX and wild-type Dular that treated at 12 °C/10 °C (day/night) were set as treatment groups, and the control groups were these two rice lines that placed in another chamber with day/night temperatures of 26 °C/22 °C. Both the treatment and the control groups for each rice line had four replicates and they were both repeated three times in the same growth chamber.

### Protein sub-localisation

The coding DNA sequence of *OsSHMT* (*LOC_Os03g52840*) was amplified and fused with *eYFP* in the pCambia2300 to construct a recombinant 35S::*OsSHMT*-*eYFP* vector for rice protoplast transformations. Subcellular localisation of *Os*SHMT was detected using confocal laser scanning microscopy. An organelle-specific protein marker (mcherry) was co-localised with OsSHMT to validate the above results. At the same time, a blank vector that contained only the yellow fluorescent protein gene was separately transferred into the rice protoplast to serve as a reference. The rice protoplasts were cultured at 28 °C for 48 h. The distribution of yellow fluorescence in the protoplast was observed by laser confocal microscopy to determine the subcellular localisation of OsSHMT protein.

### DNA pull-down fishes transcription regulators binding to the promoter of *OsSHMT*

The second-topmost leaves of the four replicates were respectively sampled at 48 h after chilling treatment. These rice leaves were quickly frozen in liquid nitrogen. The natural leaf proteins of Dular and *Lsi1*-OX were extracted using Pi-IP buffer (50 mM Tris·Cl, 150 mM NaCl, 1 mM EDTA pH 8.0, 1% Triton X-100, 1 mM PMSF, 1× EDTA-free Protease Inhibitor Cocktail, Roche, Merck) (Method S1). The promoter region (2549 bp upstream of the CDS of *OsSHMT*) was cloned from the genomic DNA of Dular, and the interacting proteins on the promoter were obtained following the protocols of our previous studies [36].

### Quantitative PCR to determine gene expression level

Total RNA from Dular and *Lsi1*-OX rice exposed to a temperature of 15 °C for 12, 24 and 36 h was extracted using Trizol and reverse-transcribed into cDNA using TransScript One-Step gDNA Removal and cDNA Synthesis SuperMix. The control groups were grown at 26 °C for 12, 24 and 36 h. Specific primers for tubulin/FtsZ domain containing protein (LOC_Os03g51600, LOC_Os05g34170, LOC_Os07g38730), histone H1 and nucleic acid binding protein (NABP) are listed in Table S3; the β-actin gene was taken as the reference. The qPCR reaction system was prepared using TransStart Tip Green qPCR SuperMix and an Eppendorf realplex^4^ instrument. The reaction process was as follows: pre-denaturation at 94 °C for 30 s, denaturation at 94 °C for 5 s, annealing at 55 °C for 15 s, extension at 72 °C for 10 s; 42 cycles. When the amplification was finished, analysing of the melting curve was conducted and specificity of the product was determined based on the melting curve. Each candidate mRNA was set with four independent replicates. The relative expression of the gene was calculated by the 2^-△△Ct^ method with the threshold cycle values (Ct) of each candidate mRNA in both the control and test samples [37].

### *Arabidopsis thaliana* transformation

The CDS of *OsSHMT* was amplified and inserted into modified pCambia3301 (with 35 s promoter) to construct the recombinant vector for *Arabidopsis thaliana* transformation using the floral dip protocols described by Clough and Bent [38]. Natural leaf proteins were extracted from a T_3_ generation homozygote of *OsSHMT* transgenic *A. thaliana* and incubated with GFP-Trap agarose (Chromotek) to collect putative interacting proteins.

### Bimolecular fluorescence complementation (BiFC) validates rice protein interactions

BiFC was conducted to validate the possible interactions between OsSHMT and the proteins identified from the Co-IP results. The genes that encode these proteins were cloned from Dular rice, construction of recombinant vectors for each gene and the subsequent protocols for BiFC are available in our previous studies [36].

### DAB staining and determination of H_2_O_2_ content

Detection of H_2_O_2_ in leaves was conducted using diaminobenzidine (DAB) staining; *OsSHMT* transgenic *A. thaliana* and its wild type were treated at 4 °C for 12, 24 and 36 h. The treatment was repeated three times in the same growth chamber. Leaves from these two lines were sampled and immersed in 50 mg/L DAB solution by vacuum-pumping for 1 h and incubated overnight at room temperature. These leaves were then decolourised with 95% ethanol in a water bath at 80 °C, and the reddish brown spots in the transgenic leaves and wild-type leaves were observed using an integrated microscope (Nikon, SMZ18). The H_2_O_2_ content in the 12 h-treated leaves of the transgenic line and the wild type were determined using an H_2_O_2_ determination kit (Solarbio Life Sciences).

### Primer sequences

A list of the primers is provided in Table S3.

## Supplementary information

**Additional file 1 Fig. S1** Subcellular localisation of *Os*SHMT protein in rice protoplast

**Additional file 2 Table S1** Sequence of *OsSHMT* gene promoter from Dular. **Table S2** Proteins interacted with OsSHMT in the *OsSHMT* transgenic *A. thaliana* using GFP-Trap Co-IP**. Table S3** Primers used in this study

**Additional file 3 Methods S1** Extraction of rice leaf protein

**Additional file 4 Electronic Supplementary Material 1**. Full length gel presents proteins binding on the OsSHMT-promoter in Dular

**Additional file 5 Electronic Supplementary Material 2.** Full length gel presents proteins binding on the OsSHMT-promoter in *Lsi1*-OX transgenic line

**Additional file 6 Electronic Supplementary Material 3.** Full length gel presents protein interactions with OsSHMT in *Arabidopsis thaliana*

## Data Availability

All data generated during this study are included in this published article and its supplementary information files, and the raw data used or analysed during the current study available from the corresponding author on reasonable request.

## Abbreviations

APX: Ascorbate peroxidase
BiFC: Bimolecular fluorescence complementation
DAB: Diaminobenzidine
ER: Endoplasmic reticulum
*Lsi1*: Low silicon gene 1
OX: Overexpression
SHMT: Serine hydroxymethyltransferase
NIP: Nodulin 26-like intrinsic protein
ROS: Reactive oxygen species
NABP: Nucleic acid binding protein
HSP70: Heat shock protein 70
