# Supplementary figures and images for: Serine hydroxymethyltransferase localised in the endoplasmic reticulum plays a role in scavenging H2O2 to enhance rice chilling tolerance
Source: BMC Plant Biol. 2020 May 26;20:236. doi: 10.1186/s12870-020-02446-9 (PMC7249644; doi:10.1186/s12870-020-02446-9)

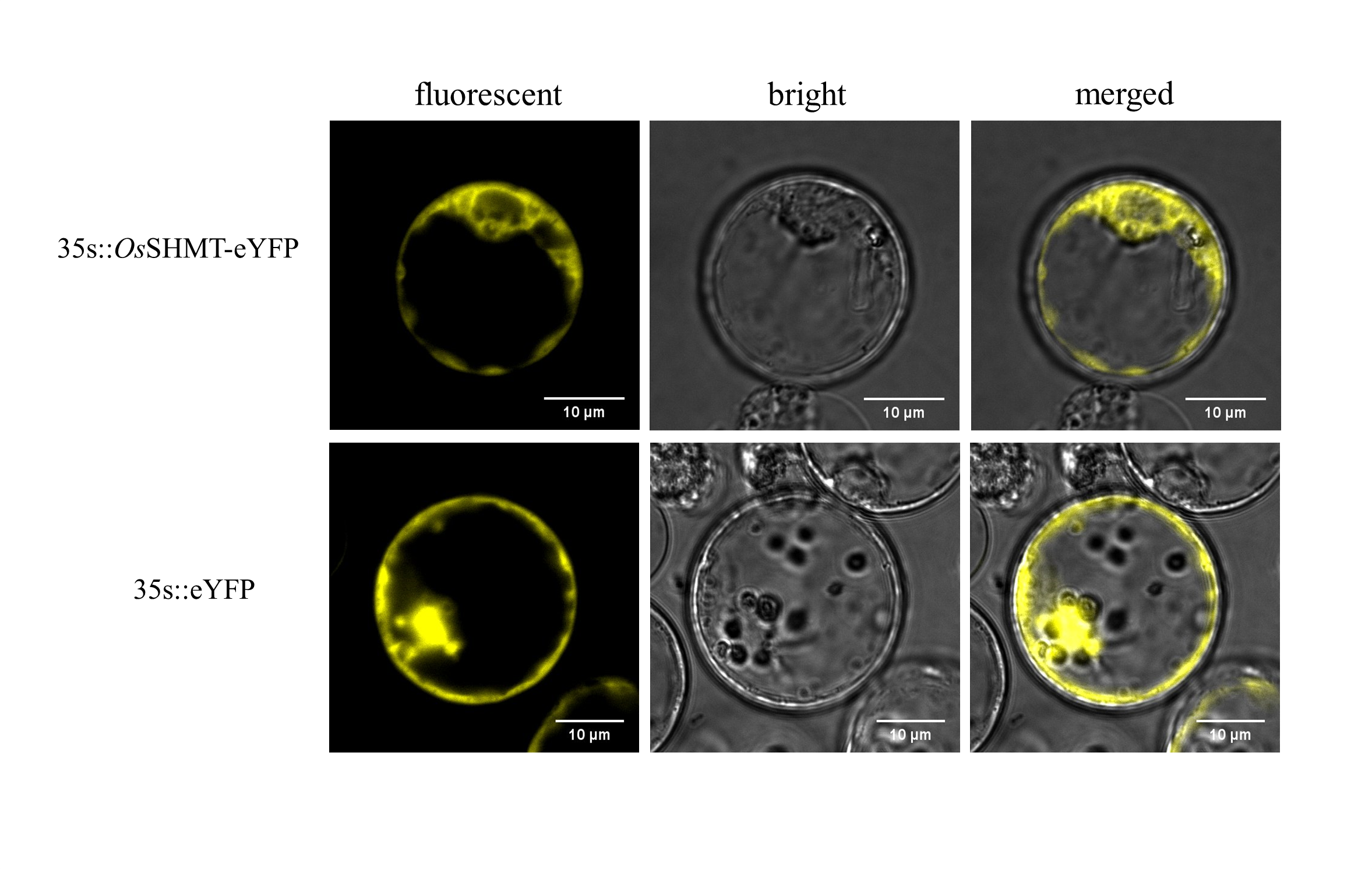

Supplement: Supplementary file 1 — Additional file 1 Fig. S1 Subcellular localisation of OsSHMT protein in rice protoplast [file 12870_2020_2446_MOESM1_ESM.tif]

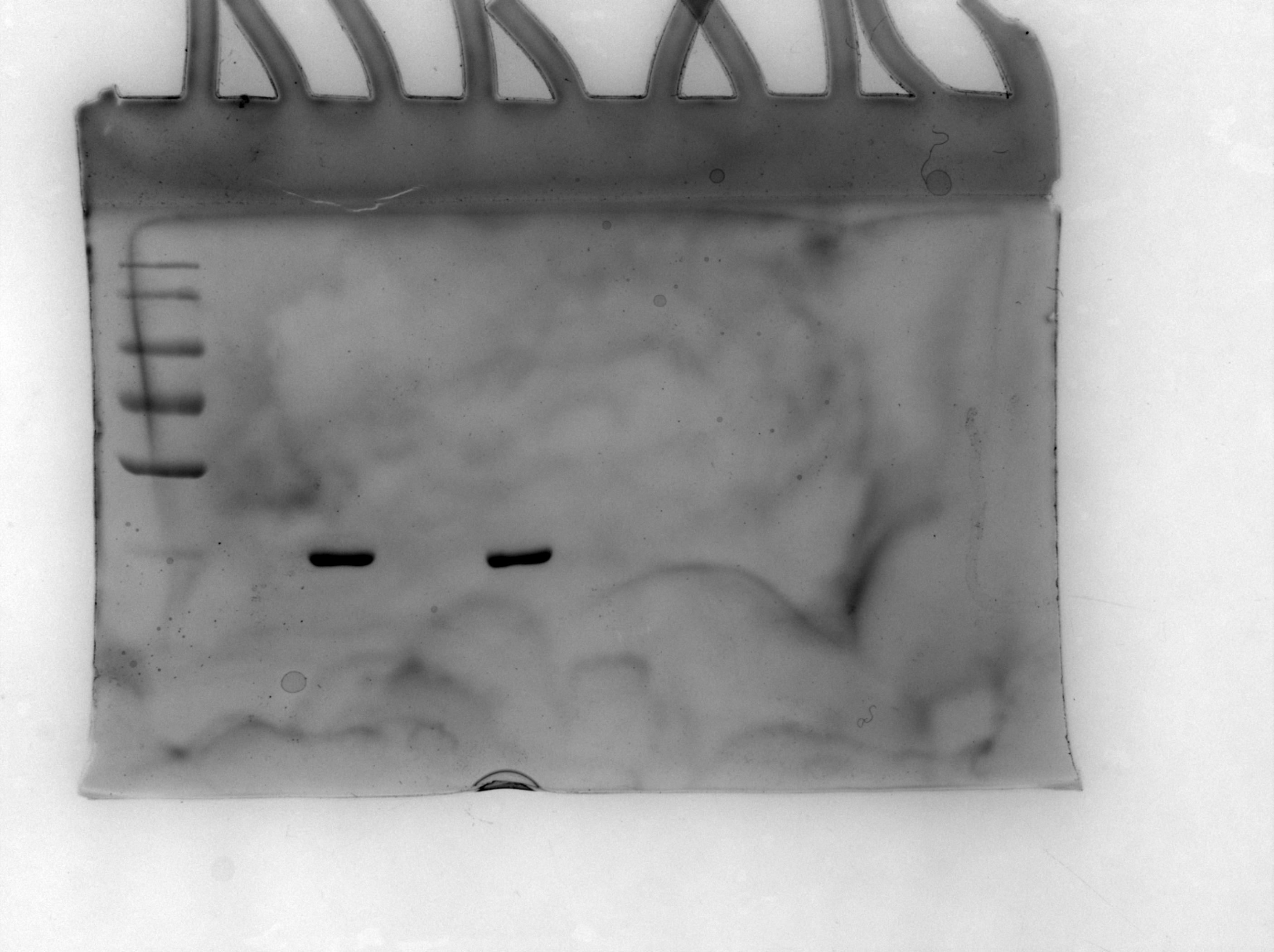

Supplement: Supplementary file 4 — Additional file 4 Electronic Supplementary Material 1. Full length gel presents proteins binding on the OsSHMT-promoter in Dular [file 12870_2020_2446_MOESM4_ESM.tif]

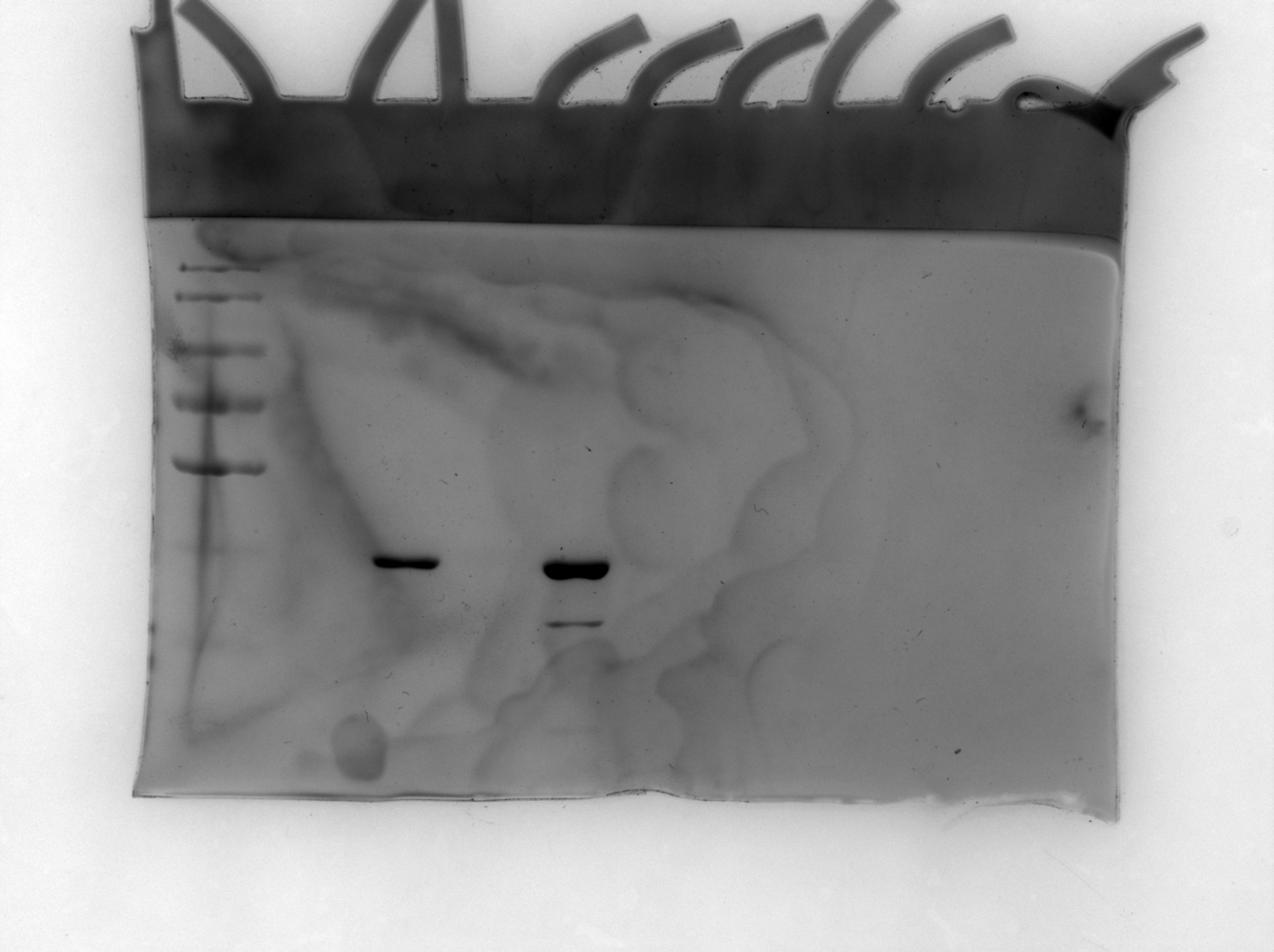

Supplement: Supplementary file 5 — Additional file 5 Electronic Supplementary Material 2. Full length gel presents proteins binding on the OsSHMT-promoter in Lsi1-OX transgenic line [file 12870_2020_2446_MOESM5_ESM.tif]

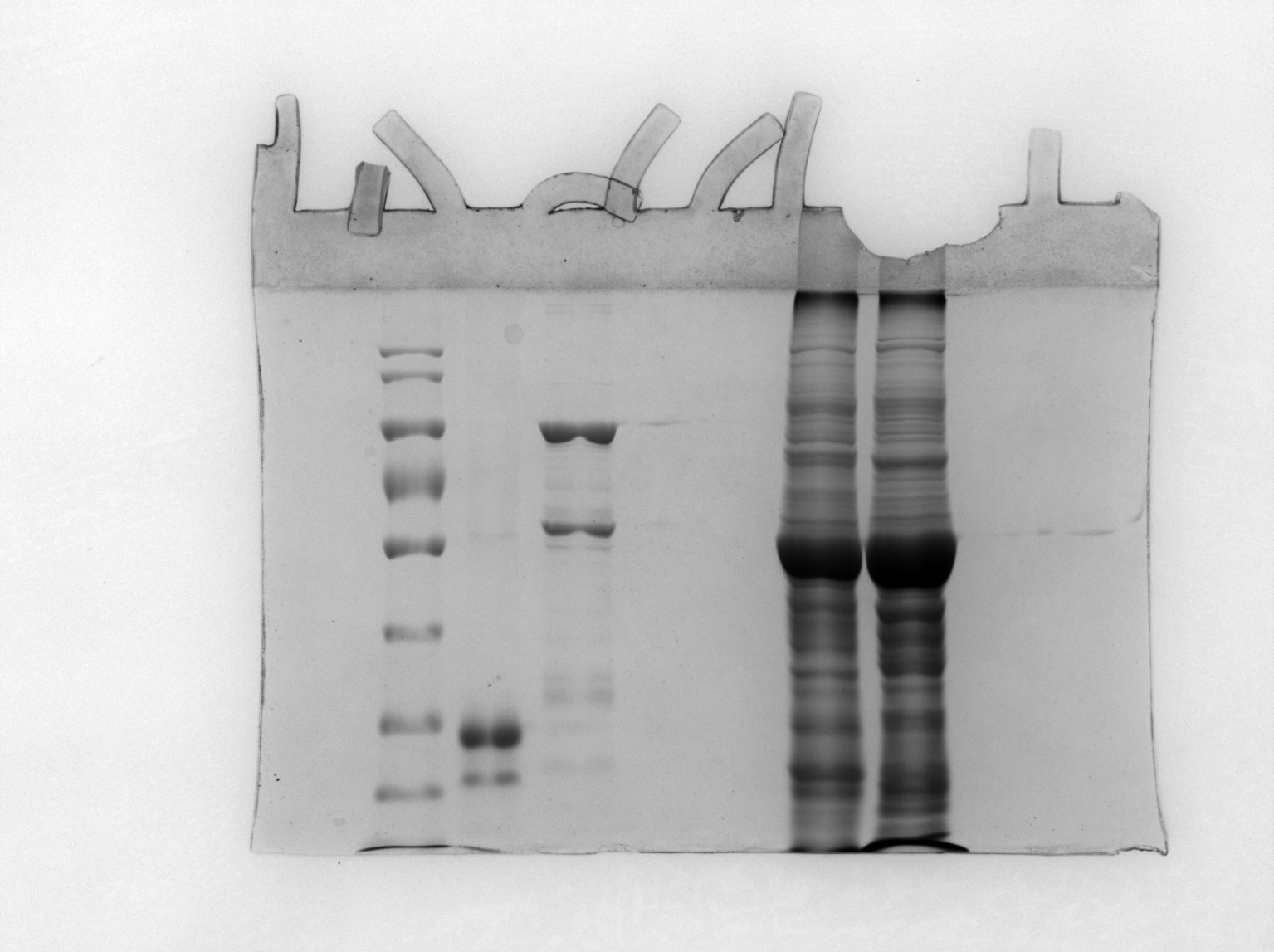

Supplement: Supplementary file 6 — Additional file 6 Electronic Supplementary Material 3. Full length gel presents protein interactions with OsSHMT in Arabidopsis thaliana [file 12870_2020_2446_MOESM6_ESM.jpg]
