# Supplementary material for: Serine hydroxymethyltransferase localised in the endoplasmic reticulum plays a role in scavenging H2O2 to enhance rice chilling tolerance
Source: BMC Plant Biol. 2020 May 26;20:236. doi: 10.1186/s12870-020-02446-9 (PMC7249644; doi:10.1186/s12870-020-02446-9)
Supplement: Supplementary file 2 — Additional file 2 Table S1 Sequence of OsSHMT gene promoter from Dular. Table S2 Proteins interacted with OsSHMT in the OsSHMT transgenic A. thaliana using GFP-Trap Co-IP. Table S3 Primers used in this study [file 12870_2020_2446_MOESM2_ESM.docx]

**Table S1 Sequence of *OsSHMT* gene promoter from Dular**

| Gene | Promoter sequence |
| --- | --- |
| *Os*SHMT | CGTGCATATGTGGATACATGCACTATCACCAACGTAATGATGCACGTTTAAATTCAAATCTTCTGTTCATTATCATGTCGTGCACAAATCATGAAGCACGTTGGACTGATAGGATAGTGCATATGCAGAGATTCATTTCTCTTTCACATAACTATAACCTTCTGTTAATACAAATCAGTTTATTCCTTTTAATATTTTCGGTTTTTTTTTTGTTGCAAGCTTTAATGACCATTTGTCGATCTTCATTTTCTAACGTACTTAGAAGTAACAGTAAAGCATAAGTGATTTACCCAAAAAATTTCTGTTTCATTTCCAGAGTTTGGACAAGACAATCCATAGTGAAAACAAGATTGCCACAGCACGTCGAGCAAAAGCAGGCTTACTGCCACAAAGAGGTTTGGTTTGCACCTGCTCTTTACTTTTTCTTCAAAACAAGGCTTAGGCTAGCTGTTTAAACATTTCAAATAAGAGTGTTCATATTTGGTCTAGCTTTACAGGGTCACCATTCACTCCATATCCTAGCAGCAAGATATGAACTGTTAAGTATGGGTAGCTATGGATGGATCCAGACAGAGCAAGGATCTGAAGTACTTTCAGCGTTTTCATGTACAATGTTCTCAGCTATACAGCAATGTATTGCAGAATTCAGGCATCGATATTTTGGGATATGGACTGGTTTTAGCAATGGTATGAACAAAAATACCCTAGCTAAGAGTTTGCAGTTCACGTGGACAGCCATGGCTTGCACTCCCAGTGGACTGAAGACTTCATGATCTTTAACAGTAAGCCGTCTTAAGAACATGAAGAGTTTTAGCAAGCACGTTTTAAAAGCACAACCAAGAAATCTGGTTCGAGTATAGTGTACATAATATTTTGCATGTACTTGTGATTTTAGAAGATTCCTACCATGACCATGTTGCATTGATGTAAAGATAGGTGTTCGACATGCAATTCTGGAAGTGGGATTCTAATTCTAAAGTGGGCAAGTATCACAGTATTTTTTTCCTCTATGCACACCTCAAATATAGCTATATTTTCGCGTGAAAAAGGGTCTGGCGATATTCAAAAGCAAAATTGGAAATTTATAGCTTGTAGAATACATGAACTCTTGCTTTCTTTCCTTGTGTTTTATTCAGATACAGATTGGTAAGAAATCTTCAAGTATATATATAGCCTTACAGCGTGACACGTTGATGATGTGCCCCGTGCTGTTAACATTGTAATTTTTTTTTATAAAAAAGAAAATCTTATCATCGTTCATAATTCCTCCCGTTTTATTGCTATTATTTGAGATGGAGATTGATCACTTTATCTGAACTCGAGTACGAACTGGTGGGCAGTGTTTGTGCTGTTGCTGACTAGTGGTCATGGAAAAGATAGGGCTCGGGTGGCCTCTGTGCACGCTGGCAGGGCAGCAATACCCTGGCCGCGTCCTCTCCATAAAACATTCAGATAAACTCAACTCCTTTTTCCCCAGCTGTTATTTTCACCATGTTTAATTTTTACCTTTGCATTTTGTATGGCCCAAGAGGCCAAGAAGCCTGTGCCTTAAGTAAATCTCCAGTAAATGTAGCGTAGTTTTTTTAATAGACAAAAATGCACCTTCTCATGTGGATAATATATTTTTGACCCCCAAAAATTGTACCAATGAGCATACTTTTATTCCTGTTATTTCTTTAATTTTCTTGGGAGAAATGTGAGATGTCGCTTTCGGAGATTAGGTGATTTTTTTTATATACACTTGCTCCCAGTGTTAGGTGATGGATGACAAAGCGTTTGTGACTTGTGATCTGTAAGTTGAGTGGTCAAGCCGCTGTTTCCTTTGCGATTTGGTACCCTGCGTCCTGATTAGTGGTAGCTAATCGCAGCAATTATATAAGCAAACCATATCTCTAAGCGCACAGGATCATTGTTTGGTTCGATACATTTTCTCCAGTGAAGAATCTTGCCTGCTTTTGGGTTGGTGGCGATCTTGAAAGCTAACACGCACGTAAAATAAACTATGCTAAATGGCAGGGCACCCGGTTAATTATTTCTTTTCCTGCCTCTGGGACCAACATCCCCGGATACTTTCAGCCATTTGATCTAGTGTCATTAGATATGGGAATATAATCTCGTTATGCGAACTTTCTCCCTTATTTTGCTGAAACCTATTTTGCTTAAATTGCACCACCCATTGCAATTTACACAATTAAAATCAAGATATTCTTTTTATCCGAATGTAACGGCATCCATAAAAAAAAAAGGTACTCTGCTATCCAAAAGAAAACGTTGTGTTGCAAAAGAATAGAAAAAACAGAAGCAGAAAAAAAAACATAAAAATAAGTAATGCTAGTGGCAAATAAGAAGAACGCGAAAACAAACGACCAATTTACATGTCTAAAATCTCAAAAAAAAAATTGGTTAAAGATTTTTTAAAAAATAAACCAAGGGTTGGAATTTTTTTTCTTTTTATTTAAAAAAAGAAGAAAATAAAAATAAAAGGAAAAGGAAAAGATTAAGCAGAGGTTGGTGATGCTGATGCGTCCGGTGTGGTGGGTGCTGGGGCTGATCACTGCAGATAAATCGCCAGCGCAAATCCACTACCTCCCAAACCC |

**Table S3 Proteins interacted with OsSHMT in the *Os*SHMT transgenic *A. thaliana* using GFP-Trap Co-IP**

| Accession | Description | Sum PEP Score | Coverage | Peptides | PSMs | Unique Peptides |
| --- | --- | --- | --- | --- | --- | --- |
| AT4G37930.1 | serine transhydroxymethyltransferase 1 | 176.46 | 52.80464 | 24 | 439 | 12 |
| AT5G17920.1 | Cobalamin-independent synthase family protein | 18.204 | 21.43791 | 12 | 13 | 5 |
| AT5G26780.3 | serine hydroxymethyltransferase 2 | 115.527 | 30.01876 | 16 | 323 | 4 |
| ATCG00490.1 | ribulose-bisphosphate carboxylases | 7.911 | 10.85595 | 4 | 7 | 4 |
| AT2G39730.1 | rubisco activase | 3.21 | 9.07173 | 3 | 3 | 3 |
| AT1G62750.1 | Translation elongation factor EFG/EF2 protein | 2.995 | 4.980843 | 3 | 3 | 3 |
| AT2G04030.1 | Chaperone protein htpG family protein | 3.373 | 3.205128 | 2 | 2 | 2 |
| AT4G13930.1 | serine hydroxymethyltransferase 4 | 1.014 | 1.698514 | 2 | 3 | 2 |
| AT4G37930.1 | serine transhydroxymethyltransferase 1 | 268.217 | 81.43133 | 42 | 575 | 28 |
| ATCG00490.1 | ribulose-bisphosphate carboxylases | 59.448 | 45.30271 | 21 | 71 | 21 |
| ATCG00480.1 | ATP synthase subunit beta | 39.443 | 50.60241 | 19 | 35 | 18 |
| AT5G26780.1 | serine hydroxymethyltransferase 2 | 166.679 | 59.96132 | 30 | 314 | 16 |
| ATCG00120.1 | ATP synthase subunit alpha | 30.137 | 26.82446 | 13 | 28 | 11 |
| AT1G07940.1 | GTP binding Elongation factor Tu family protein | 13.637 | 25.61247 | 9 | 12 | 9 |
| AT4G32520.2 | serine hydroxymethyltransferase 3 | 14.589 | 16.44612 | 8 | 10 | 8 |
| AT4G13940.1 | S-adenosyl-L-homocysteine hydrolase | 7.569 | 12.78351 | 6 | 6 | 6 |
| AT1G20620.1 | catalase 3 | 10.604 | 16.66667 | 7 | 7 | 5 |
| AT2G28000.1 | chaperonin-60alpha | 6.781 | 11.09215 | 5 | 5 | 5 |
| AT3G46780.1 | plastid transcriptionally active 16 | 6.212 | 12.15686 | 5 | 5 | 5 |
| AT2G07698.1 | ATPase, F1 complex, alpha subunit protein | 7.527 | 6.177606 | 5 | 7 | 3 |
| AT1G23310.1 | glutamate:glyoxylate aminotransferase | 5.898 | 7.276507 | 3 | 3 | 3 |
| AT1G04820.1 | tubulin alpha-4 chain | 5.585 | 10 | 3 | 3 | 3 |
| AT3G29360.1 | UDP-glucose 6-dehydrogenase family protein | 4.418 | 7.5 | 3 | 3 | 3 |
| AT5G44340.1 | tubulin beta chain 4 | 3.6 | 8.108108 | 3 | 3 | 3 |
| AT4G37930.1 | serine transhydroxymethyltransferase 1 | 205.363 | 61.5087 | 25 | 241 | 14 |
| AT5G23060.1 | calcium sensing receptor | 40.773 | 46.25323 | 14 | 16 | 14 |
| ATCG00490.1 | ribulose-bisphosphate carboxylases | 27.376 | 30.48017 | 13 | 21 | 13 |
| AT5G42270.1 | FtsH extracellular protease family | 25.602 | 28.125 | 12 | 16 | 12 |
| AT4G10340.1 | light harvesting complex of photosystem II 5 | 40.747 | 51.07143 | 10 | 20 | 10 |
| AT2G39730.1 | rubisco activase | 29.38 | 24.89451 | 9 | 12 | 9 |
| AT1G13440.1 | glyceraldehyde-3-phosphate dehydrogenase C2 | 25.007 | 31.36095 | 8 | 10 | 8 |
| AT3G08580.1 | ADP/ATP carrier 1 | 10.969 | 22.83465 | 8 | 11 | 8 |
| AT1G78900.1 | vacuolar ATP synthase subunit A | 10.961 | 16.21188 | 8 | 9 | 8 |
| AT1G07890.2 | ascorbate peroxidase 1 | 9.281 | 37.6 | 7 | 8 | 7 |
| AT2G30950.1 | FtsH extracellular protease family | 23.004 | 21.58273 | 12 | 15 | 6 |
| ATCG00020.1 | photosystem II reaction center protein A | 17.298 | 20.96317 | 6 | 9 | 6 |
| AT1G71500.1 | Rieske (2Fe-2S) domain-containing protein | 15.276 | 27.87456 | 6 | 9 | 6 |
| AT5G19760.1 | Mitochondrial substrate carrier family protein | 7.573 | 24.16107 | 6 | 7 | 6 |
| AT5G26780.3 | serine hydroxymethyltransferase 2 | 101.84 | 34.89681 | 16 | 152 | 5 |
| AT5G02490.1 | Heat shock protein 70 (Hsp 70) family protein | 15.568 | 9.035222 | 5 | 7 | 5 |
| AT1G61520.1 | photosystem I light harvesting complex gene 3 | 12.322 | 22.71062 | 5 | 5 | 5 |
| AT1G07940.1 | GTP binding Elongation factor Tu family protein | 6.559 | 14.2539 | 5 | 6 | 5 |
| AT3G01500.2 | carbonic anhydrase 1 | 32.305 | 42.93948 | 10 | 21 | 4 |
| ATCG00120.1 | ATP synthase subunit alpha | 10.003 | 10.45365 | 4 | 5 | 4 |
| AT3G63410.1 | S-adenosyl-L-methionine-dependent methyltransferases superfamily protein | 9.624 | 18.93491 | 4 | 4 | 4 |
| AT5G65010.2 | asparagine synthetase 2 | 6.685 | 8.635579 | 4 | 4 | 4 |
| ATCG00680.1 | photosystem II reaction center protein B | 6.58 | 12.40157 | 4 | 4 | 4 |
| ATCG00270.1 | photosystem II reaction center protein D | 6.545 | 15.29745 | 4 | 4 | 4 |
| AT2G21330.1 | fructose-bisphosphate aldolase 1 | 6.479 | 10.27569 | 4 | 4 | 4 |
| AT5G46800.1 | Mitochondrial substrate carrier family protein | 5.09 | 10 | 4 | 4 | 4 |
| AT5G14740.1 | carbonic anhydrase 2 | 31.403 | 41.99396 | 9 | 19 | 3 |
| AT3G08940.2 | light harvesting complex photosystem II | 19.349 | 33.79791 | 7 | 15 | 3 |
| AT1G06430.1 | FTSH protease 8 | 18.01 | 15.91241 | 9 | 10 | 3 |
| AT5G01530.1 | light harvesting complex photosystem II | 14.137 | 28.27586 | 7 | 9 | 3 |
| ATCG00480.1 | ATP synthase subunit beta | 6.277 | 8.634538 | 3 | 3 | 3 |
| AT2G04842.1 | threonyl-tRNA synthetase, putative | 6.145 | 5.846154 | 3 | 3 | 3 |
| ATCG00540.1 | photosynthetic electron transfer A | 5.917 | 11.5625 | 3 | 3 | 3 |
| AT3G53580.1 | diaminopimelate epimerase family protein | 5.788 | 9.392265 | 3 | 3 | 3 |
| AT4G20360.1 | RAB GTPase homolog E1B | 5.117 | 8.193277 | 3 | 3 | 3 |
| AT5G20010.1 | RAS-related nuclear protein-1 | 4.75 | 14.47964 | 3 | 3 | 3 |
| AT3G10670.1 | non-intrinsic ABC protein 7 | 3.964 | 12.72189 | 3 | 3 | 3 |

**Table S3 Primers used in this study**

|  | Gene | Forward primer (5’-3’) | Reverse primer (5’-3’) |
| --- | --- | --- | --- |
| Protein localization | *p2300-OsSHMT* | CGACTCTAGAGGATCCATGGCCATGGCGACGGCGCTC | CCATACTAGTGGATCCGTTCTTGTACTTCATGGTTTCTT |
|  | *p2300-OsLsi1* | CGACTCTAGAGGATCCATGGCCAGCAACAACTCG | CTCGGTACCCGGGGATCCCACTTGGATGTTCTCCATC |
|  | *Ubi-1301-GFP* | GTCGACTCTAGAGGATCCATGGTGAGCAAGGGCGAGGA | CTCGGTACCCGGGGATCCTCCGGACTTGTACAGCTCGTCC |
|  | *1301-OsLsi1* | GGATCCATGGCCAGCAACAACTCG | GGTACCTCACACTTGGATGTTCTCCATC |
| 1. *thaliana*   transformation | *OsSHMT* | TCCAGCTCCAGGATCCATGGCCATGGCGACGGCGCTC | GAGAAAGCTTGGATCCTTAGTTCTTGTACTTCATGGTTT |
| *Promoter region of OsSHMT*  (For DNA – pull down) | *OsSHMT* | (5’ biotin labeled ) CGTGCATATGTGGATACATGCA | (5’ biotin labeled ) GGGTTTGGGAGGTAGTGGATTC |
| EMSA | *-2077~-1641* | TAGCAGCAAGATATGAACTGTTAAG | AGAATTGCATGTCGAACACCTAT |
|  | *-1640~-1199* | AGAATTGCATGTCGAACACCTAT | GGAAGTGGGATTCTAATTCTAAAGT |
|  | *-1198~-757* | GCACGCTGGCAGGGCAGCAATA | CAGGGTACCAAATCGCAAAGGAAAC |
|  | *-756~-315* | CGTCCTGATTAGTGGTAGCTAATCG | TTGCAACACAACGTTTTCTTTTGG |
|  | *-314~-1* | AAGAATAGAAAAAACAGAAGCAG | GGGTTTGGGAGGTAGTGGATTC |
| qPCR | *OsSHMT* | AGATACTACGGTGGAAACGAAT | TCAATAGGGCAGTGTAAACATG |
|  | *OsAAA-ATPase* | CGGCAGACATCATCAAGAAGG | TGTTCATCAGGGTGGCGTTC |
|  | *LOC_Os03g51600* | GTTGCTACCGCCAGCTCTT | TTGCCATAGTCCACAGAAAGG |
|  | *LOC_Os05g34170* | TGCGACTGCCTCCAAGGTTTC | TGAGCATCATCCTGTCCGGGTAC |
|  | *LOC_Os07g38730* | AAGGAGGATGCCGCTAACA | AAGCAAGGAGTGGGTGGAG |
|  | *Oshistone H1* | GCGAAGGAGGCTTTGGAGGC | CGAAGTAGGGCGGGTGGTGT |
|  | *OsNABP* | GCAGGCAATCAGCAAATC | CTTCAATAGGTTCCCAGACA |
|  | *β-actin* | CTGCGGGTATCCATGAGACT | GCAATGCCAGGGAACATAGT |
| BiFC | *OsSHMT* | ATGGCGCGCCACTAGTATGGCGATGGCTTCGCACCACC | CACCTCCTCCACTAGTATCCTTGTACTTCATGTCGGATACC |
|  | *OsATP-synα* | ATGGCGCGCCACTAGTATGGCAACCCTTCGAGTCGACGA | CACCTCCTCCACTAGTAAGGGAAAACCGTTCGAGTTGTTCC |
|  | *OsATP-synβ* | ATGGCGCGCCACTAGTATGGCGACTCGCCGGGCCCTCT | CACCTCCTCCACTAGTTGAAGCCGACTCCTTGGCGATC |
|  | *OsHSP70* | ATGGCGCGCCACTAGTATGGCGGCGTCGCTGCTTCTCC | CACCTCCTCCACTAGTCTTCTTGACCTCCTCATACTCAGCC |
|  | *OsMSCP* | ATGGCGCGCCACTAGTATGGCGGCGTCCCCGTCCGCT | CACCTCCTCCACTAGTTGCAGCTTCCTCAACTTCTGATC |
|  | *OsAXP* | ATGGCGCGCCACTAGTATGGCTAAGAACTACCCCGTCGTG | CACCTCCTCCACTAGTAGCATCAGCGAACCCCAGTTCGGA |
| expression of recombinant proteins in *Escherichia coli* | *Os*AAA-ATPase | GGCTGATATCGGATCCATGGGTGAATTCTGTGGTGAGG | GCTCGAATTCGGATCCACCGTAGAAGGAACCAGTCTTC |
|  | LOC_Os03g51600 | GGCTGATATCGGATCCATGAGGGAGTGCATCTCGATCC | GCTCGAATTCGGATCCGTACTCGTCACCATCATCGCCA |
|  | LOC_Os05g34170 | GGCTGATATCGGATCCATGAGGGAGATCTTGCACATCC | GCTCGAATTCGGATCCCTCCTGTATCGCGTCCTCTTCT |
|  | LOC_Os07g38730 | GGCTGATATCGGATCCATGAGAGAGATCATCAGCATCCA | CTCGAATTCGGATCCATAGTCTTCTCCATCGTCGTTCT |
|  | *Os*histone H1 | GGCTGATATCGGATCCATGGCGACTGCGACCGAGGA | GCTCGAATTCGGATCCGGCGCTGGCCTTGTTGGCC |
|  | *Os*NABP | GGCTGATATCGGATCCATGGACCGGTACCAGAGGGT | GCTCGAATTCGGATCCGTTTCCTCGTCCACCACGGC |

Note: the sequence underlined indicates the specific vector sequence for gene infusion
