# Supplementary material for: Serine hydroxymethyltransferase localised in the endoplasmic reticulum plays a role in scavenging H2O2 to enhance rice chilling tolerance
Source: BMC Plant Biol. 2020 May 26;20:236. doi: 10.1186/s12870-020-02446-9 (PMC7249644; doi:10.1186/s12870-020-02446-9)
Supplement: Supplementary file 3 — Additional file 3 Methods S1 Extraction of rice leaf protein [file 12870_2020_2446_MOESM3_ESM.docx]

**Protocols of rice leaf protein extraction**

1) Grind tissues in liquid nitrogen into fine powder.

2) Estimate volume of pulverized tissue powder; add 2 times volume of the freshly prepared Pi-IP buffer.

3) Votex well and let the mixture thaw on ice or rotate at 4℃ for 15 min

4) Centrifuge the samples at 4℃, 16,000×g, for 15 min in a centrifuge.

5) Pour supernatant into a new tube, using sterile Miracloth to filter the supernatant. Repeat the centrifugation step to ensure removal of material that pellets at 16,000×g.

Components of Pi-IP buffer :

| Component (stock solution) | Final concentration |
| --- | --- |
| 0.5M Tris, pH 7.5 | 50 mM |
| 5M NaCl | 150 mM |
| 0.5M EDTA, pH 8.0 | 1 mM |
| 20% Triton X-100 | 1 % |
| 100mM PMSF | 1 mM |
| 100×EDTA-free Protease Inhibitor Cocktail | 1× |
